# Soluble CD59 is a Novel Biomarker for the Prediction of Obstructive Chronic Lung Allograft Dysfunction After Lung Transplantation

**DOI:** 10.1038/srep26274

**Published:** 2016-05-24

**Authors:** Kevin Budding, Eduard. A. van de Graaf, Tineke Kardol-Hoefnagel, Johanna M. Kwakkel-van Erp, Bart D. Luijk, Erik-Jan D. Oudijk, Diana A. van Kessel, Jan C. Grutters, C. Erik Hack, Henderikus G. Otten

**Affiliations:** 1 Laboratory of Translational Immunology, University Medical Center Utrecht, Utrecht, The Netherlands; 2 Department of Respiratory Medicine, University Medical Center Utrecht, Utrecht, The Netherlands; 3 Center of Interstitial Lung Diseases, St Antonius Hospital, Nieuwegein, The Netherlands; 4 Departments of Rheumatology and Dermatology, University Medical Center Utrecht, Utrecht, The Netherlands

## Abstract

CD59 is a complement regulatory protein that inhibits membrane attack complex formation. A soluble form of CD59 (sCD59) is present in various body fluids and is associated with cellular damage after acute myocardial infarction. Lung transplantation (LTx) is the final treatment for end-stage lung diseases, however overall survival is hampered by chronic lung allograft dysfunction development, which presents itself obstructively as the bronchiolitis obliterans syndrome (BOS). We hypothesized that, due to cellular damage and activation during chronic inflammation, sCD59 serum levels can be used as biomarker preceding BOS development. We analyzed sCD59 serum concentrations in 90 LTx patients, of whom 20 developed BOS. We observed that BOS patients exhibited higher sCD59 serum concentrations at the time of diagnosis compared to clinically matched non-BOS patients (*p* = 0.018). Furthermore, sCD59 titers were elevated at 6 months post-LTx (*p* = 0.0020), when patients had no BOS-related symptoms. Survival-analysis showed that LTx patients with sCD59 titers ≥400 pg/ml 6 months post-LTx have a significant (*p* < 0.0001) lower chance of BOS-free survival than patients with titers ≤400 pg/ml, 32% vs. 80% respectively, which was confirmed by multivariate analysis (hazard ratio 6.2, *p* < 0.0001). We propose that circulating sCD59 levels constitute a novel biomarker to identify patients at risk for BOS following LTx.

CD59 is a membrane anchored complement regulatory protein that inhibits membrane attack complex (MAC) formation, thereby preventing complement mediated cell lysis^1^,^2^. Regulation of CD59 expression plays a pivotal role in various diseases. Hyperexpression of CD59 on endothelial cells increases resistance to complement mediated cell damage, which has been proposed to explain the process known as graft accommodation to circulating pathogenic allo- or autoantibodies following organ transplantation^3^,^4^,^5^. In contrast, age-related macular degeneration is associated with lower CD59 expression on monocytes^6^, whereas paroxysmal nocturnal hemoglobinuria is caused by CD59 deficiency^7^.

Different processes, including cell damage and activation, induce the release of membrane anchored proteins from the cell surface, a process designated as shedding^8^. CD59 can detach from the cell membrane to be released into the circulation or the interstitial fluid in a soluble form (sCD59). Indeed, sCD59 can be detected in various body fluids including urine, milk, serum, and plasma^9^,^10^,^11^. Various studies have designated sCD59 as biomarker for disease activity. Elevated circulating sCD59 concentrations have been found in acute myocardial infarction^10^, and higher serum titers of glycated sCD59 have been described in diabetes mellitus^12^. Despite these findings, the role of sCD59 and its association with disease is still largely unidentified.

Lung transplantation (LTx) is the final treatment for selected patients with end-stage lung diseases. Long-term outcome after LTx is hampered by chronic lung allograft dysfunction (CLAD) which can be divided into an obstructive CLAD (bronchiolitis obliterans syndrome, BOS) and a restrictive CLAD (restrictive allograft syndrome, RAS). Clinical characterization of BOS involves obstructive pulmonary function tests (forced expiratory volume in one second (FEV_1_) below 80% from baseline FEV_1_) enduring for more than three weeks. The second form of CLAD, RAS, is identified by restrictive pulmonary functions tests (forced vital capacity (FVC) below 80% baseline FVC) presenting more than 3 weeks^13^. BOS is considered to be caused by chronic rejection which will lead to obliterative bronchiolitis and lung damage. In the clinical setting the FEV_1_ decrease is used as surrogate marker and will occur after obliterative bronchiolitis has already developed. The recognition of this damage and therefore the diagnosis obliterative bronchiolitis prior to BOS development may be of help in designing therapies for BOS prevention. Five years after LTx the BOS-free survival is 50%, though most patients who survive short-term complications, will eventually develop BOS^14^.

Considering high CD59 expression by bronchial epithelial cells and sCD59 release following cellular damage^2^,^10^, we hypothesized that sCD59 may be a marker for inflammatory lung tissue damage predicting BOS incidence and progression. Therefore, we measured sCD59 in serum samples of LTx patients and assessed the correlation of sCD59 titers with BOS incidence. We show, that sCD59 levels measured six months post-LTx are strongly associated with BOS development and thus may be used as clinical marker for chronic rejection after LTx.

## Results

### Patient demographics

Eighty-nine patients treated with LTx because of chronic obstructive pulmonary disease (COPD, n = 40), cystic fibrosis (CF, n = 30), interstitial lung disease (ILD, n = 18); including extrinsic allergic alveolitis (n = 1), idiopathic pulmonary fibrosis (n = 6), lymphangioleiomyomatosis (n = 3), non-specific interstitial pneumonia (n = 1), pulmonary Langerhans cell histiocytosis (n = 2), progressive systemic sclerosis (n = 2) and sarcoidosis (n = 3)) or pulmonary hypertension (n = 1), were included in the study, and 20 healthy controls (HC). Further demographic details are given in Table 1. Twenty patients developed BOS, diagnosed according to international guidelines^15^ and RAS was not observed. No differences were found between BOS and non-BOS patients, except episodes of acute rejection.

### Concentrations of serum sCD59 are elevated at the time of BOS diagnosis

**Figure 1:**
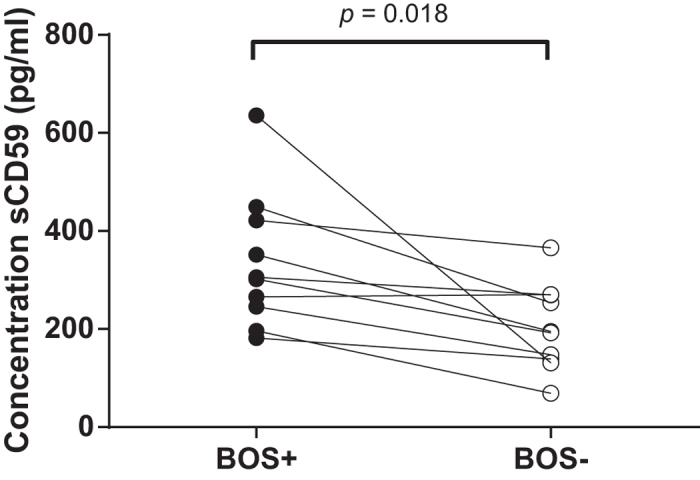
Serum sCD59 concentrations are elevated at the time of BOS diagnosis. From 10 BOS (filled circles) and 10 non-BOS (open circles) patients serum sCD59 concentrations were measured (matched pairs indicated by lines). BOS patients show significant higher sCD59 titers at the time of BOS diagnosis (335.5 ± 43.31 pg/ml vs. 203.3 ± 27.59 pg/ml) compared to their matched counterparts (*p* = 0.018, Gaussian distribution, paired *t*-test).

We first examined, in a pilot experiment, whether sCD59 levels differed between BOS and non-BOS patients at the time of BOS diagnosis. To this end we measured sCD59 in serum of 10 BOS patients at the month of BOS diagnosis, and compared these levels with those in 10 non-BOS patients matched for gender, age, primary disease, and month post-LTx. BOS was diagnosed at month 35 post-LTx on average (Supplementary Table 1). BOS patients have significant higher sCD9 concentrations compared to their matched counterpart (*p* = 0.018, data normally distributed, paired *t*-test) at the time of diagnosis (Fig. 1). In total, 80% of the BOS patients have elevated sCD59 serum concentrations compared to the median of matched controls.

### Serum sCD59 concentrations are elevated before BOS diagnosis

**Figure 2:**
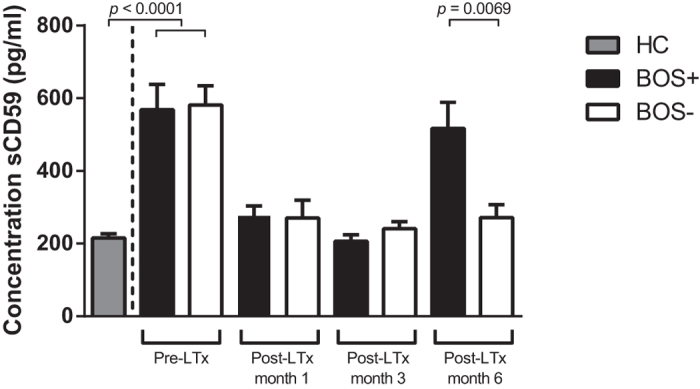
Serum sCD59 concentrations are elevated before the clinical diagnosis of BOS. sCD59 serum concentrations were measured pre-LTx and at fixed time points after LTx in 10 matched BOS (black bars) and non-BOS patients (white bars), the normally distributed data are depicted as mean ± SEM. Pre LTx, all patients presented with significantly increased sCD59 titers compared to healthy controls (grey bar). Post-LTx all patients presented with sCD59 serum concentrations comparable to healthy controls. No differences were observed either at 1 month or 3 months post-LTx. BOS patients show significant higher serum concentrations sCD59 (517.0 ± 72.22 pg/ml vs. 271.8 ± 35.22 pg/ml) 6 months post-LTx compared to their matched counterparts, before the clinical diagnosis of BOS (*p* = 0.0069, Unpaired *t*-test).

To study potential sCD59 concentration differences before BOS diagnosis we analyzed sCD59 concentrations in the same matched cohort of ten patients at fixed time points after transplantation, and examined the course of sCD59 levels in either group. At month 1 and 3 post-LTx serum concentrations of sCD59 were comparable in both patients groups. However, a significant difference (*p* = 0.0069) was observed at month 6 post-LTx (Fig. 2, Gaussian distribution, mean ± SEM, unpaired *t*-test). These elevated sCD59 titers persisted over time until BOS diagnosis (data not shown).

Also, we analyzed sCD59 serum concentrations in healthy controls and compared them to sCD59 serum titers end-stage lung disease patients, determined in samples drawn prior to LTx procedure. End-stage lung disease patients presented with significant increased sCD59 concentration compared to HC (577.0 ± 268.1 vs. 215.8 ± 49.9 respectively, *p* < 0.0001). No difference was observed between patients who would eventually develop BOS versus those who do not, based on pre-LTx sCD59 levels (Fig. 2).

### sCD59 levels at 6 months post-LTx are indicative for BOS incidence

**Figure 3:**
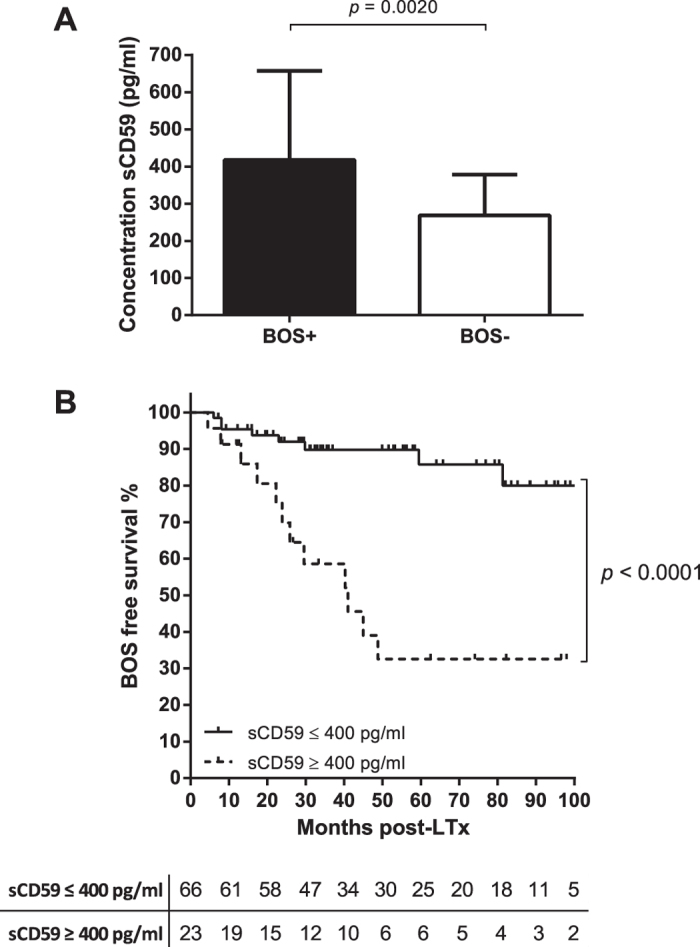
sCD59 serum titers 6 months post-LTx are indicative for BOS incidence. (**A**) Whole cohort analysis shows that sCD59 titers, displayed in pg/ml, 6 months post-LTx are elevated in BOS patients (*n* = 20, black bar) compared to non-BOS patients (*n* = 69, white bar), *p* = 0.0020, Mann Whitney test. Both median and interquartile range are depicted due to the fact that the non-BOS serum titers follow a non-Gaussian distribution. (**B)** Death-censored Kaplan-Meier analysis for BOS incidence after LTx. LTx patients who present serum sCD59 titers above 400 pg/ml 6 months post-LTx (dashed line) have a significant lower chance of BOS free survival than patients with titers below 400 pg/ml (closed line), 32% vs. 80% respectively, *p* < 0.0001.

To verify that sCD59 titers are elevated at month 6 post-LTx in BOS compared to non-BOS patients, we examined serum concentrations of sCD59 in an additional patient cohort. Analysis of sera taken 6 months post-LTx from 10 additional BOS and 59 non-BOS patients confirmed the prior observation (data not shown). Taken these results together, total analysis of 89 LTx patients, 20 BOS and 69 non-BOS resulted in a significant difference (*p* = 0.0020, non-Gaussian distribution, median + interquartile range, Mann-Whitney test) in sCD59 titers 6 months post-LTx (Fig. 3A). A ROC curve was used to determine the possibility of predicting BOS 6 months post-LTx. This analysis demonstrated the optimal cut-off value for positivity at 400 pg/ml, AUC 0.72 (0.58–0.86) with a sensitivity of 60% and a specificity of 84% (Supplementary Figure 1). A Kaplan-Meier analysis showed that a sCD59 titer above 400 pg/ml significantly (*p* < 0.0001) reduced likelihood of BOS-free survival compared to a sCD59 titer below 400 pg/ml, 32% vs. 80% respectively (Fig. 3B). Furthermore, we assessed the possibility of confounding by usage of a multivariate Cox proportional hazard model including known risk factors as treated acute rejection, defined as a decline in lung function in absence of other causes of lung function decline treated with methylprednisolone pulse, high donor age and as patient being at high risk for CMV reactivation^16^. This model identified sCD59 titers ≥400 pg/ml 6 months post-LTx as an independent predictor for BOS development after transplantation (hazard ratio 6.2, 95% CI 2.3–14.3, *p* < 0.0001), see Table 2.

We also assessed the relation between sCD59 and other parameters. The type of transplantation (single vs. bilateral) did no correlate with sCD59 titers or LTx outcome. Club cell secretory protein 16 (CC16), an epithelial cell damage and leakage marker^17^, measured 6 months post-LTx, did not correlate with sCD59 titers. Also, we found no increase of general markers of inflammation such as C reactive protein (CRP) or neutrophil counts specifically in the BOS group. Finally, since sCD59 can be secreted via glomerular filtration, we investigated whether patients with increased sCD59 levels had a worse renal function. No differences in creatinine levels were found between patients with elevated or normal sCD59 (data not shown).

## Discussion

Long-term survival post-LTx is severally hampered by BOS development. Current diagnostic tools for BOS upon LTx are limited to FEV_1_ measurement and there is a clinical need for biomarkers to early identify patients at risk for developing BOS. We evaluated serum concentrations of a soluble form of the membrane anchored complement regulatory protein CD59, sCD59, as a risk marker for BOS in a cohort of LTx patients. We show that serum levels of sCD59 in LTx patients are increasing prior to BOS diagnosis. This indicates that sCD59 titers may be used to identify patients at risk for BOS development earlier.

This study is limited with respect to the number of patients diagnosed with BOS after LTx. Therefore, we could not correlate sCD59 titers with different stages of BOS. Furthermore, the number of cases presented, limits the inclusion of covariates in our multivariate analysis. Hence, validation of these results is desired using a multicenter approach to increase patient numbers.

Compared to the ISHLT registry report^14^, we observer a lower incidence of BOS in our study population. Overall the 5-year incidence of BOS in our center is close to 70%, which is 20% higher compared to ISHLT figures. Deviations from these numbers have been reported previously^18^ and could be attributed to overall shorter follow-up time in our cohort, intensity of post-LTx follow up, and patient compliance.

Absolute serum levels of sCD59 do differ comparatively with previous studies. Landi *et al*. measured serum concentrations of sCD59 in adults and children and found mean titers of approximately 70 ng/ml^19^. Väkevä *et al*. found sCD59 concentrations in healthy controls to be around 8 pg/ml, though these measurements have been performed in plasma^10^. Ghosh *et al*. quantified sCD59 in standard peptide units, so no comparison could be made with our data^12^. Reported differences in sCD59 serum titers between the previous and our studies are probably reflecting differences in antibodies and standards used for the assays. Standardization of methods to measure sCD59 is urgently needed. We measured sCD59 with a commercial ELISA which is widely available to the medical community.

sCD59 is detectable in body fluids including plasma, urine, tears, sweat and human milk^1^,^2^,^20^. sCD59 exists with or without the phospholipid tail, and both variants differ in complement inhibiting capacities^9^. Our assay could not discriminate the two isoforms. Several mechanisms have been proposed to explain the occurrence of circulatory sCD59, including shedding via secretion of membrane vesicles containing phospholipid-tailed sCD59, or enzymatic cleavage via phospholipases C and/or D. The latter cut GPI anchored proteins at their phospholipid tail resulting in anchor-less sCD59. Other mechanisms claim that a substantial MAC deposition may lead to CD59 release, or that lipid secretion results in CD59 shedding from the cell surface^9^,^19^. Zangh *et al*. have shown that activation of vascular endothelial cells leads to an increase in phospholipase C secretion, which mediates sCD59 release^21^. This mechanism has also been proposed for diabetes mellitus, where enzymatic shedding in chronically activated endothelial cells is supposed to explain increased glycated sCD59 concentrations in serum^12^,^22^. Our data do not allow conclusions regarding the mechanisms underlying increased sCD59 levels in BOS or non-BOS LTx patients. However, diabetes was no confounder for sCD59 levels in our transplantation cohort.

CD59 is a GPI anchored membrane protein that suppresses complement mediated cell lysis via MAC inhibition^1^,^22^,^23^,^24^. CD59 is highly expressed on vascular endothelial cells. Also, expression is observed in the ductal epithelia of salivary systems and the bronchi^2^. Human respiratory tract tissue, including bronchi and alveolar tissue, expresses high levels of complement regulatory proteins, and most predominantly CD59. In healthy tissue, CD59 expression is observed at the apical and luminal side of the epithelium, whereas during inflammation a more diffuse and basolateral distribution is observed^25^. Interestingly, evidence suggests increased complement activation in bronchoalveolar lavage fluid (BALF) of patients with various lung diseases including cystic fibrosis^26^ and sarcoidosis^27^. Infections and local inflammatory reactions may lead to increased complement activation and upregulation of complement regulatory proteins in order to protect self-tissue from complement mediated cell damage. Indeed, CD59 expression is increased in diseased compared to healthy tissue and CD59 is also distributed along the extracellular matrix of the respiratory tract^25^. The same pattern of increased complement regulation during inflammation has been observed in the gut. CD59 is upregulated in patients with gastritis, coeliac disease and inflammatory bowel disease. It is proposed that cytokines secreted in inflammatory lesions can induce CD59 expression on the apical surface of colonic epithelium^28^.

We demonstrate that sCD59 increases at the time BOS becomes clinically manifest, and that sCD59 levels at month 6 post-LTx discriminate patients with BOS from those without. These elevated sCD59 levels do not seem to be protective against aggravated complement activity, presumably due to decreased functionality^9^. The explanation that sCD59 reflected renal clearance was ruled out since we found no correlation with creatinine levels. We therefore assume that sCD59 reflects the inflammatory processes ensuing in the transplanted lungs preceding the clinical manifestations of BOS. To discriminate whether cellular damage or activation underlies increased sCD59, we conducted a correlation analysis between CC16, a lung epithelial injury marker^17^, and sCD59, but no correlation was observed. We found no relation between sCD59 and general markers of infection, including CRP and neutrophil counts. Generally, these results suggest that sCD59 reflects a local inflammatory process that is linked to the development of BOS rather than a general state of immune activation.

We hypothesize a model in which chronic inflammation, a hallmark of BOS development, results in cellular activation, presumably of endothelial or epithelial cells, and increased expression of CD59 which is then shed from the cell surface and detectable at increased levels in the circulation. Our observations are in concordance with previous results, where decreased membrane attached CD59 and increased sCD59 was observed in patients after acute myocardial infarction^10^. Interestingly, both endothelial and epithelial cells when activated secrete IL-8, CCL-5 and CCL2, measured in BALF, cytokines associated with BOS development^29^, strengthening the concept of the role of cellular activation in the BOS pathogenesis. Unfortunately, in our study we did not have access to BALF since surveillance bronchoscopy was not performed. Therefore, we could not quantify sCD59 in BALF or correlate any changes in cytokine secretion or cellular subset composition in BALF to sCD59 titers.

Recently, novel biomarkers have been identified to predict BOS development. Also, auto- and alloantibodies have been associated with BOS incidence^4^. It is therefore reasonable to assume that humoral rejection and complement activation plays a pivotal role in BOS pathogenesis, as shown by Magro *et al*.^5^. Furthermore, soluble CD30 and TARC are found to have a predictive value on BOS development^30^,^31^. Further studies are needed to establish the predictive value of each of these markers for BOS.

To summarize, we report a significant increase of sCD59 in patients with BOS following LTx. This increase occurred at 6 months post-LTx and preceded clinical manifestations of BOS. Further studies are needed to elucidate the molecular mechanisms underlying increased sCD59 in BOS. We propose that sCD59 may provide a suitable biomarker to identify patients at risk for BOS development after LTx.

## Patients and Methods

### Patients

Eighty-nine patients that underwent LTx between September 2003 and May 2011 at the Heart Lung Center of the Utrecht Medical Center were included in the study, based upon serum availability, drawn at month 6 post-LTx (55% of the transplanted patients within this time-period, Supplementary Figure 2). From all patients informed consent was obtained and the study was approved by the Medical Ethical Committee of the University Medical Center Utrecht (METC 06–144). All methods were carried out in accordance with the approved guidelines. Blood samples were collected several hours prior to transplantation, monthly during the first year of follow up, and every 3 months thereafter. Blood samples were processed and stored as serum aliquots at −80 °C. Patients were treated with an immunosuppressive regime, consisting of tacrolimus, basiliximab, prednisone, and mycophenolate mofetil. Patients at risk for CMV reactivation (defined as CMV- recipient/CMV + donor) were treated with valganciclovir until 6 months post-LTx.

### Assay for soluble CD59

sCD59 concentrations in serum were determined via ELISA (USCN Life Science Inc., China) according to manufacturer’s instructions. Serum samples were thawed, diluted 1:400 in PBS, and incubated on NUNC maxisorp plates (NUNC, Roskilde, Denmark) coated with a mouse anti-human CD59 monoclonal antibody for 2 hours at 37 °C. The plates were washed and incubated with biotin-conjugated rabbit anti-human CD59 polyclonal antibodies for 1 hour at 37 °C. Plates were washed again and incubated for 30 minutes with streptavidin-horseradish peroxidase (HRP). Bound biotinylated antibodies were visualized with TMB substrate for 15 minutes at 37 °C. The reaction was terminated with H_2_SO_4_. Optical density of the wells was measured at 450 nm with a Multiskan EX Microplate photometer (ThermoScientific, IL). OD450 values were compared to standard concentrations of recombinant CD59 and are expressed in pg/ml. The minimal detectable dose of human sCD59 is 6.7 pg/ml. Intra- and inter-assay coefficients of variation are <10% and <12% respectively. Furthermore, the recovery rate of the ELISA in our hands was 87% (range 83–91%), which was in concordance with the manufacturers description (average recovery rate in serum 87%, range 80–94%). All samples were measured in duplicate.

### Statistics

Statistical analysis was performed using GraphPad Prism version 5.03 (GraphPad Software Inc., San Diego, CA) and SPSS version 20 (IBM Corp., Armonk, NY). Distribution of data was tested for normality via the D’Agostino & Pearson omnibus normality test. Data following a Gaussian distribution are represented as mean value ± SEM, whereas data not normally distributed are displayed as median and interquartile range, indicated in the respective results section and the corresponding figure legend. Categorical data such as age, gender, and primary disease were analyzed using the Fischer’s exact test, type of transplantation using the Pearson’s χ^2^ test. Differences between continuous variables were tested via ANOVA. Differences between 2 groups were tested with unpaired *t* test in case the values followed a Gaussian distribution, and with the Mann-Whitney test otherwise. BOS-free survival was analyzed using a death censored, i.e. graft failure definition excludes patient death with a functioning graft, Kaplan-Meier analysis. Finally, we used a Cox proportional hazards model to identify sCD59 as an independent predictor for BOS incidence. A *p*-value < 0.05 was considered to be statistical significant.

## Additional Information

**How to cite this article**: Budding, K. *et al*. Soluble CD59 is a Novel Biomarker for the Prediction of Obstructive Chronic Lung Allograft Dysfunction After Lung Transplantation. *Sci. Rep.* **6**, 26274; doi: 10.1038/srep26274 (2016).

## Supplementary Material

Supplementary Information

## Figures and Tables

**Table 1:** Clinical and demographic profile of lung transplantation patients.

	Total LTx patients		*p*-value
	BOS	Non BOS	
Total number	20	69	
BOS grade			
I	8	N.A.	
II	5	N.A.	
III	7	N.A.	
Onset of BOS (month)	28 (5–81)	N.A.	
Mean follow up (months)	48 (10–105)	50 (7–118)	0.72
Type of transplantation			0.38
Single	3	15	
Bilateral	17	54	
Mean age (years)	45 (16–63)	45 (17–63)	0.91
Gender			0.053
Male	6	37	
Female	14	32	
Primary disease			0.72
COPD	10	30	
CF	7	23	
ILD	3	15	
PH	0	1	
Episode of acute rejection	9	13	*0.015*

Patients are clustered by diagnosis, which are COPD (chronic obstructive pulmonary disease), CF (cystic fibrosis), ILD (interstitial lung disease), and PH (pulmonary hypertension). Significant intergroup differences are given in italics.

**Table 2:** Multivariate analysis on BOS incidence after LTx.

	Hazard ratio (95% CI)	*p-*value
sCD59 ≥ 400 pg/ml	6.2 (2.4–15.8)	*<0.0001*
Donor age (≥60)	4.6 (1.4–15.0)	*0.011*
CMV reactivation	0.7 (0.3–2.1)	0.572
Episode of acute rejection	1.8 (0.7–4.4)	0.230

Within our multivariate analysis we included potential confounders for BOS incidence after LTx (see results section 3.4). In our cohort both a sCD59 titer ≥400 pg/ml and donor age ≥60 were identified as independent predictors for BOS development. Significant intergroup differences are given in italics.

## References

[b1] Davies A. . CD59, an LY-6-like protein expressed in human lymphoid cells, regulates the action of the complement membrane attack complex on homologous cells. J. Exp Med. 170, 637–54 (1989). 2475570 10.1084/jem.170.3.637 PMC2189447

[b2] Meri S., Waldmann H. & Lachmann P. J. Distribution of protectin (CD59), a complement membrane attack inhibitor, in normal human tissues. Lab Invest. 65, 532–7 (1991). 1721667

[b3] Sacks S. H. & Zhou W. The role of complement in the early immune response to transplantation. Nat Rev Immunol. 12, 431–42 (2012). 22627861 10.1038/nri3225

[b4] Hachem R. R. . Antibodies to K-alpha 1 tubulin and collagen V are associated with chronic rejection after lung transplantation. Am J Transplant 12, 2164–71 (2012). 22568593 10.1111/j.1600-6143.2012.04079.x PMC3409301

[b5] Magro C. M., Ross P. Jr., Kelsey M., Waldman W. J. & Pope-Harman A. Association of humoral immunity and bronchiolitis obliterans syndrome. Am J Transplant 3, 1155–66 (2003). 12919096 10.1034/j.1600-6143.2003.00168.x

[b6] Singh A. . Altered expression of CD46 and CD59 on leukocytes in neovascular age-related macular degeneration. Am J Ophthalmol 154, 193–9 (2012). 22541656 10.1016/j.ajo.2012.01.036

[b7] Nevo Y. . CD59 deficiency is associated with chronic hemolysis and childhood relapsing immune-mediated polyneuropathy. Blood 121, 129–35 (2013). 23149847 10.1182/blood-2012-07-441857

[b8] Hayashida K., Bartlett A. H., Chen Y. & Park P. W. Molecular and cellular mechanisms of ectodomain shedding. Anat Rec (Hoboken) 293, 925–37 (2010). 20503387 10.1002/ar.20757 PMC4621804

[b9] Meri S., Lehto T., Sutton C. W., Tyynela J. & Baumann M. Structural composition and functional characterization of soluble CD59: heterogeneity of the oligosaccharide and glycophosphoinositol (GPI) anchor revealed by laser-desorption mass spectrometric analysis. Biochem J. 316 (Pt 3), 923–35 (1996). 8670172 10.1042/bj3160923 PMC1217438

[b10] Vakeva A., Lehto T., Takala A. & Meri S. Detection of a soluble form of the complement membrane attack complex inhibitor CD59 in plasma after acute myocardial infarction. Scand J Immunol. 52, 41–41 (2000). 10.1046/j.1365-3083.2000.00783.x 11013013

[b11] Hakulinen J. & Meri S. Shedding and enrichment of the glycolipid-anchored complement lysis inhibitor protectin (CD59) into milk fat globules. Immunology 85, 495–501 (1995). 7558140 PMC1383925

[b12] Ghosh P. . A specific and sensitive assay for blood levels of glycated CD59: a novel biomarker for diabetes. Am J Hematol. 88, 670–6 (2013). 23670858 10.1002/ajh.23478 PMC3775575

[b13] Verleden G. M., Raghu G., Meyer K. C., Glanville A. R. & Corris P. A new classification system for chronic lung allograft dysfunction. J Heart Lung Transplant 33, 127–33 (2014). 24374027 10.1016/j.healun.2013.10.022

[b14] Christie J. D. . The Registry of the International Society for Heart and Lung Transplantation: 29th adult lung and heart-lung transplant report-2012. J Heart Lung Transplant 31, 1073–86 (2012). 22975097 10.1016/j.healun.2012.08.004

[b15] Estenne M. . Bronchiolitis obliterans syndrome 2001: an update of the diagnostic criteria. J Heart Lung Transplant. 21, 297–310 (2002). 11897517 10.1016/s1053-2498(02)00398-4

[b16] Hennessy S. A. . Donor factors are associated with bronchiolitis obliterans syndrome after lung transplantation. Ann Thorac Surg. 89, 1555–62 (2010). 20417777 10.1016/j.athoracsur.2010.01.060 PMC3033807

[b17] Kropski J. A., Fremont R. D., Calfee C. S. & Ware L. B. Clara Cell Protein (CC16), a Marker of Lung Epithelial Injury, Is Decreased in Plasma and Pulmonary Edema Fluid From Patients With Acute Lung Injury. Chest. 135, 1440–7 (2009). 19188556 10.1378/chest.08-2465 PMC2716712

[b18] Verleden G. M. . Survival determinants in lung transplant patients with chronic allograft dysfunction. Transplantation. 92, 703–8 (2011). 21836537 10.1097/TP.0b013e31822bf790

[b19] Landi A. P. . Determination of CD59 protein in normal human serum by enzyme immunoassay, using octyl-glucoside detergent to release glycosyl-phosphatidylinositol-CD59 from lipid complex. Immunol Lett. 90, 209–13 (2003). 14687727 10.1016/j.imlet.2003.07.001

[b20] Bjorge L. . Soluble CD59 in pregnancy and infancy. Immunol Lett 36, 233 (1993). 7688713 10.1016/0165-2478(93)90058-a

[b21] Zhang L. . Lipopolysaccharide activated phosphatidylcholine-specific phospholipase C and induced IL-8 and MCP-1 production in vascular endothelial cells. J. Cell Physiol. 226, 1694–701 (2011). 21413027 10.1002/jcp.22500

[b22] Zhang J., Gerhardinger C. & Lorenzi M. Early complement activation and decreased levels of glycosylphosphatidylinositol-anchored complement inhibitors in human and experimental diabetic retinopathy. Diabetes 51, 3499–504 (2002). 12453906 10.2337/diabetes.51.12.3499

[b23] Meri S. . Human protectin (CD59), an 18,000-20,000 MW complement lysis restricting factor, inhibits C5b-8 catalysed insertion of C9 into lipid bilayers. Immunology 71, 1–9 (1990). 1698710 PMC1384213

[b24] Rollins S. A. & Sims P. J. The complement-inhibitory activity of CD59 resides in its capacity to block incorporation of C9 into membrane C5b-9. J Immunol 144, 3478–83 (1990). 1691760

[b25] Varsano S., Frolkis I. & Ophir D. Expression and distribution of cell-membrane complement regulatory glycoproteins along the human respiratory tract. Am J Respir Crit Care Med. 152, 1087–93 (1995). 7545058 10.1164/ajrccm.152.3.7545058

[b26] Fick R. B. Jr, Robbins R. A., Squier S. U., Schoderbek W. E. & Russ W. D. Complement activation in cystic fibrosis respiratory fluids: *in vivo* and *in vitro* generation of C5a and chemotactic activity. Pediatr Res. 20, 1258–68 (1986). 3540828 10.1203/00006450-198612000-00014

[b27] Hamacher J., Sadallah S., Schifferli J. A., Villard J. & Nicod L. P. Soluble complement receptor type 1 (CD35) in bronchoalveolar lavage of inflammatory lung diseases. Eur Respir J. 11, 112–9 (1998). 9543279 10.1183/09031936.98.11010112

[b28] Berstad A. E. & Brandtzaeg P. Expression of cell membrane complement regulatory glycoproteins along the normal and diseased human gastrointestinal tract. Gut, 42, 522–9 (1998). 9616315 10.1136/gut.42.4.522 PMC1727075

[b29] Reynaud-Gaubert M. . Upregulation of chemokines in bronchoalveolar lavage fluid as a predictive marker of post-transplant airway obliteration. J Heart Lung Transplant 21, 721–30 (2002). 12100898 10.1016/s1053-2498(02)00392-3

[b30] Fields R. C. . Elevated soluble CD30 correlates with development of bronchiolitis obliterans syndrome following lung transplantation. Transplantation 82, 1596–1601 (2006). 17198242 10.1097/01.tp.0000241076.46033.4c

[b31] Paantjens A. W. . Serum thymus and activation regulated chemokine levels post-lung transplantation as a predictor for the bronchiolitis obliterans syndrome. Clin Exp Immunol 154, 202–8 (2008). 18785972 10.1111/j.1365-2249.2008.03764.x PMC2612724

