# Supplementary material for: Soluble CD59 is a Novel Biomarker for the Prediction of Obstructive Chronic Lung Allograft Dysfunction After Lung Transplantation
Source: Sci Rep. 2016 May 24;6:26274. doi: 10.1038/srep26274 (PMC4877647; doi:10.1038/srep26274)
Supplement: Supplementary Information [file srep26274-s1.pdf]

Soluble CD59 is a Novel Biomarker for the Prediction of Obstructive Chronic Lung Allograft Dysfunction After Lung Transplantation

Kevin Budding[1], Eduard. A. van de Graaf[2], Tineke Kardol-Hoefnagel[1], Johanna M. Kwakkel-van Erp[2], Bart D. Luijk[2], Erik-Jan D. Oudijk[3], Diana A. van Kessel[3], Jan C. Grutters[3], Cornelis E. Hack[1,4], and Henderikus G. Otten[1]

[1]Laboratory of Translational Immunology, University Medical Center Utrecht, Utrecht, The Netherlands

[2]Department of Respiratory Medicine, University Medical Center Utrecht, Utrecht, The Netherlands,

[3]Center of Interstitial Lung Diseases, St Antonius Hospital, Nieuwegein, The Netherlands,

[4]Departments of Rheumatology and Dermatology, University Medical Center Utrecht, Utrecht, The Netherlands

Corresponding author

K. Budding

Laboratory of Translational Immunology

University Medical Center Utrecht

F.03.821

P.O. Box 85500

3508 GA Utrecht

Tel: +31 88 75 59 024

Fax: +31 30 25 17 107

Mail: k.budding@umcutrecht.nl

Supplementary Table 1: Clinical and demographic profile of matched BOS and non-BOS patients

Patients are divided in BOS and non-BOS groups. Patients were matched for gender, age, onset of BOS and primary disease. No significant differences were observed between the two patient groups.

Supplementary Figure 1: ROC curve analysis of sCD59 concentrations

A ROC curve was generated to identify the optimal concentration of sCD59 as prognostic marker for BOS development. This analysis demonstrated the optimal cut-off value for positivity at 400 pg/ml, AUC 0.72 (0.58–0.86) with a sensitivity of 60% and a specificity of 84%.

Supplementary Figure 2: CONSORT type chart of patient inclusion

A total of 162 patients were transplanted between September 2003 and May 2011. Seventy-three patients were excluded leaving 89 patients eligible for analysis.

Supplementary Table 1. Matched BOS and non-BOS patients

	Total LTx patients		
	**BOS**	**Non BOS**	***p* value**
**Total number**	10	10	
**Gender**			0.709
Male	2	2	
Female	8	8	
**Mean age** (years)	42 (16 - 61)	43 (21 - 61)	0.957
**Onset of BOS** (months)	35 (23 - 59)	n.a.	n.a.
**Primary disease**			
COPD	6	6	0.675
CF	4	4	

BOS: bronchiolitis obliterans syndrome; COPD: chronic obstructive pulmonary disease;
CF: cystic fibrosis

Supplementary Figure 1

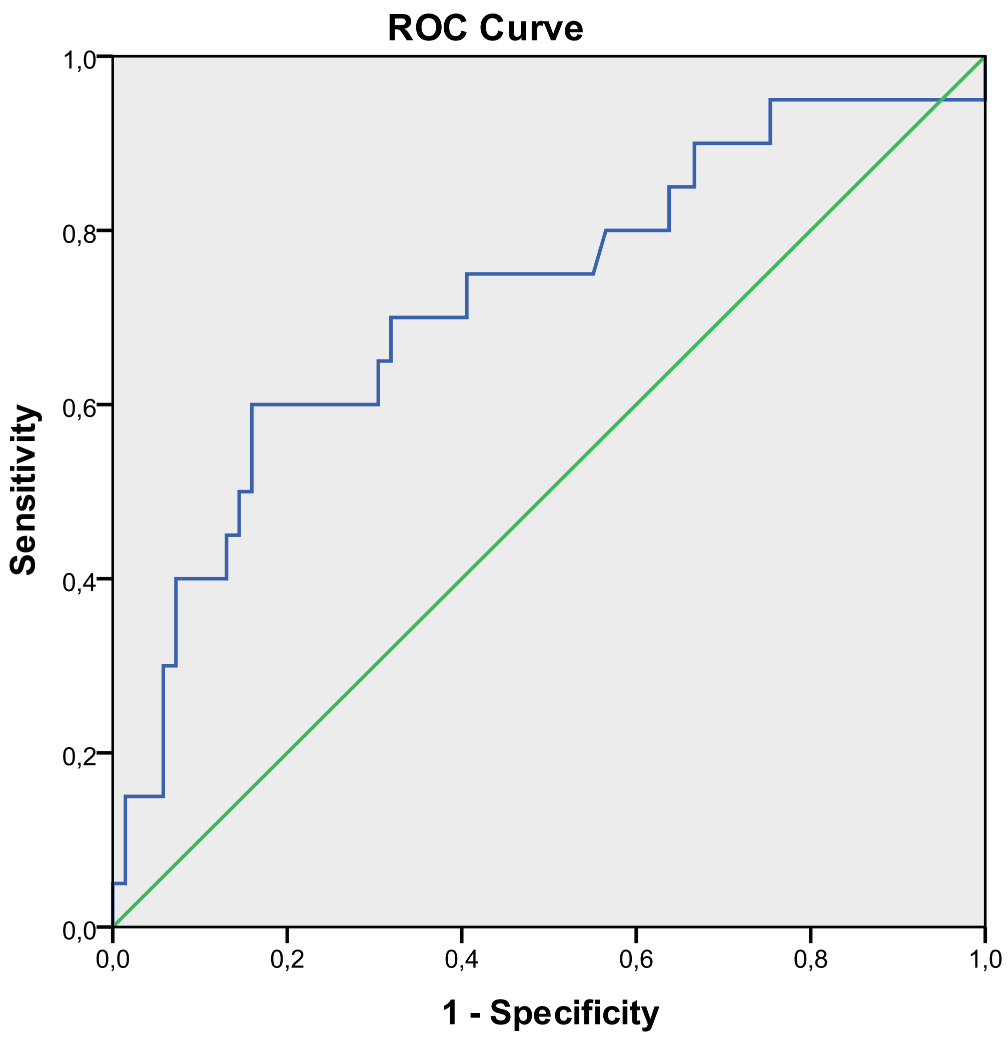

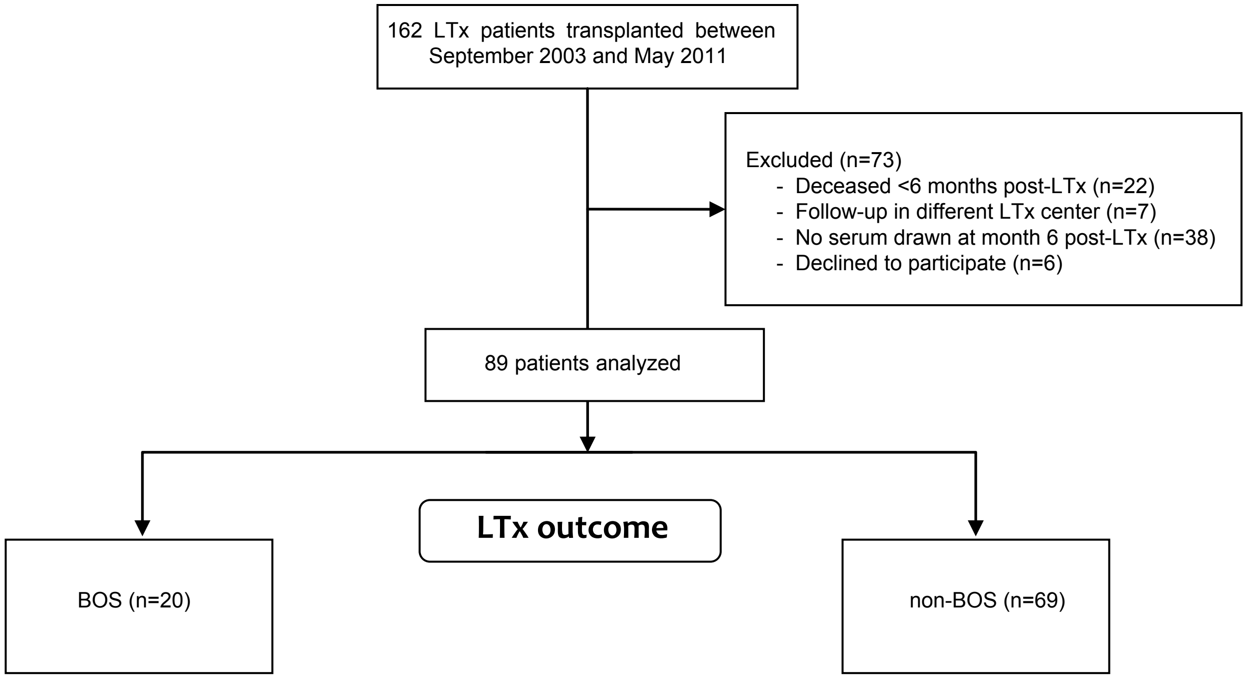
162 LTx patients transplanted between September 2003 and May 2011
Excluded (n=73)
- Deceased <6 months post-LTx (n=22)
- Follow-up in different LTx center (n=7)
- No serum drawn at month 6 post-LTx (n=38)
- Declined to participate (n=6)
89 patients analyzed
LTx outcome
BOS (n=20)
non-BOS (n=69)
